# Experimental evidence of impacts of an invasive parakeet on foraging behavior of native birds

**DOI:** 10.1093/beheco/aru025

**Published:** 2014-03-07

**Authors:** Hannah L. Peck, Henrietta E. Pringle, Harry H. Marshall, Ian P.F. Owens, Alexa M. Lord

**Affiliations:** ^a^Division of Ecology and Evolution, Silwood Park, Imperial College London, Ascot SL5 7PY, UK,; ^b^Institute of Zoology, Zoological Society of London, London NW1 4RY, UK, and; ^c^The Natural History Museum, London SW7 5BD, UK

**Keywords:** alien, ecological impacts, foraging behavior, interspecific interference competition, parrot, ringnecked parakeet.

## Abstract

Resource competition is one potential behavioral mechanism by which invasive species can impact native species, but detecting this competition can be difficult due to the interactions that variable environmental conditions can have on species behavior. This is particularly the case in urban habitats where the disturbed environment can alter natural behavior from that in undisturbed habitats. The rose-ringed parakeet (*Psittacula krameri*), is an increasingly common invasive species, predominantly associated with large urban centers. Using an experimental approach, we tested the behavioral responses of native garden birds in response to the presence of a rose-ringed parakeet versus the presence of a similarly sized and dominant native bird, the great spotted woodpecker (*Dendrocopos major*). Parakeet presence significantly reduced feeding rates and increased vigilance among native birds compared with our control treatments. Of visits made by native birds in the presence of a parakeet, feeding was more likely to occur in sites within the parakeet range compared with sites outside, suggesting some habituation of native birds has occurred following prior exposure to parakeets but overall foraging behavior is still disrupted. The results of our study suggest that nonnative species can have complex and subtle impacts on native fauna and show that a nonnative competitor can impact native species simply through their presence near resources.

## Lay Summary

Invasive rose-ringed parakeets caused behavioral changes in native garden birds that reduced their feeding rates. Understanding how invasive species impact native species can be complex, especially in urban environments where many other factors are also at play. We therefore used an experiment to disentangle these factors and demonstrate that parakeets are more disruptive than a dominant native competitor.

## INTRODUCTION

It is well documented that nonnative species can have devastating impacts on biodiversity (Mack et al. 2000; Simberloff 2005; Shochat et al. 2010; Vilà et al. 2010) but despite extensive research, a high degree of uncertainty still exists with regard to the mechanisms of these impacts on native fauna (Perrings et al. 2002; Vilà et al. 2010). Anecdotal evidence and a lack of understanding of the nature and dynamics of the invasion process have contributed to this uncertainty (Mack et al. 2000; Gurevitch and Padilla 2004). Knowledge of the behavioral impacts resulting from nonlethal interspecific competition between native and nonnative species can, therefore, give insight into the mechanisms and consequences of invasions (reviewed in Chapple et al. 2012).

It has been hypothesized that both native and nonnative species which are abundant in urban areas are those that are most adaptable and able to exploit resources in a disturbed environment (McKinney 2006). These “urban adapter” and “urban exploiter” species tend to have broad diets and high behavioral flexibility (Sol 2002). These characteristics are likely to increase their potential to compete for food with a variety of species, particularly at supplementary feeding stations (Shochat et al. 2010) and displace other species from urban habitats (e.g., Parsons et al. 2006). For instance, increased interspecific competition can result in reduced foraging success of the affected individuals, increased time spent on vigilance, displacement to less high value resources and ultimately result in nonlethal fitness consequences with the potential to indirectly affect population level changes (Cresswell 2008). Interspecific competition is widely thought to play a role in the impact of nonnative species (Probert and Litvaitis 1996; Kiesecker et al. 2001; Wauters et al. 2002; Dame et al. 2006; Soares and Serpa 2006) but may be difficult to demonstrate unambiguously (Blackburn et al. 2009) perhaps in part because the ecological impacts of nonnative species can be difficult to distinguish from other potential environmental causes (Shochat et al. 2006; Dures and Cumming 2010). It is thought that nonnative species that displace native species are likely to be better able to exploit resources and thus have strong interference effects on native species (Amarasekare 2002). There is thus a need for experimental investigation to identify how interspecific interference might lead to behavioral changes driven by nonnative species (Dame et al. 2006; Strubbe et al. 2011).

Another factor that requires consideration when studying the potential impact of invasive organisms on the behavior of native species is habituation. It has been suggested that simply the novelty of the invasive species may disrupt normal behavior in native species, by eliciting a neophobic avoidance response (McEvoy et al. 2008). Neophobic impacts can be reduced or altered by repeated exposure to the novel species, as shown in cases of habituation in predator–prey interactions (reviewed by Brown and Chivers 2005). It has also been found that bird populations, which establish in urban areas, tend to have higher tolerance for novelty due to higher behavioral flexibility and reduced neophobia (Martin and Fitzgerald 2005; Levey et al. 2009; Evans et al. 2010; Lowry et al. 2013). This could mean that birds in urban areas, which share food sources with invasive species, may not be affected as much as bird populations outside urban areas due to a preadaption to cope with an altered environment including altered species presence. However, there has been relatively little work investigating habituation of native species to invasive species and the effects of prior exposure on competitive interactions (but see Webb et al. 2008; Abril and Gómez 2009; Nelson et al. 2011). In this context, rapid ongoing range expansion by exotic populations therefore offer the opportunity to investigate whether native species do habituate to the presence of invasive species (Freidenfelds et al. 2012).

In this study, we test for evidence of interference competition with native species and for habituation in the native species to an urban population of the rose-ringed parakeet. This nonnative species is listed as one of the top 100 most invasive alien species in Europe (Vilà et al. 2009) and is a common invasive bird species around the world (Feare 1996), particularly in urban areas (Strubbe and Matthysen 2009). This study system is ideal because, although locations of establishment of invasive bird populations have been well recorded (Duncan et al. 2003), there is little quantitative evidence of their impacts on native faunas (Blackburn et al. 2009; Strubbe et al. 2011).

The high-density populations of the rose-ringed parakeet in urban centers, provides a situation in which interspecific competition for resources with native species might be expected (Tayleur 2010). Urban gardens and parks provide alternative food sources such as supplemental feed and nonnative plant species. As such they enable rapid population growth of both native and nonnative adapter species, reflected in their positive association with human population density (Daniels and Kirkpatrick 2006; McKinney 2006; Fuller et al. 2008; Strubbe and Matthysen 2009).

Previous studies on rose-ringed parakeets have been limited to investigating competition for nest sites and have suggested that the impact of this form of competition is likely to be negligible (Strubbe et al. 2010; Czajka et al. 2011; Newson et al. 2011). However, the parakeet’s highly varied diet (Feare 1996), its ability to eat nonnative plants and human-provided food sources (Koutsosms et al. 2001), as well as the fact that individuals have been found to spend half their feeding time at artificial bird feeders (Clergeau and Vergnes 2011), means that it has potential to compete with a wide variety of native bird species for these resources and particularly with species found in urban gardens.

Here, we use an experimental approach to address the following questions: 1) Does the presence of a nonnative competitor at a high value food source alter the foraging behavior of native species? 2) Does the native species’ response to the presence of the nonnative competitor differ from that of a similarly dominant native species with which they have coexisted? 3) Is the strength of response to the presence of an establishing nonnative competitor correlated with prior exposure to the nonnative species? 4) If so, is this response indicative of reinforcement of avoidance behavior of the nonnative species, or of habituation to the nonnative species?

## MATERIALS AND METHODS

### Experiment sites

**Figure 1:**
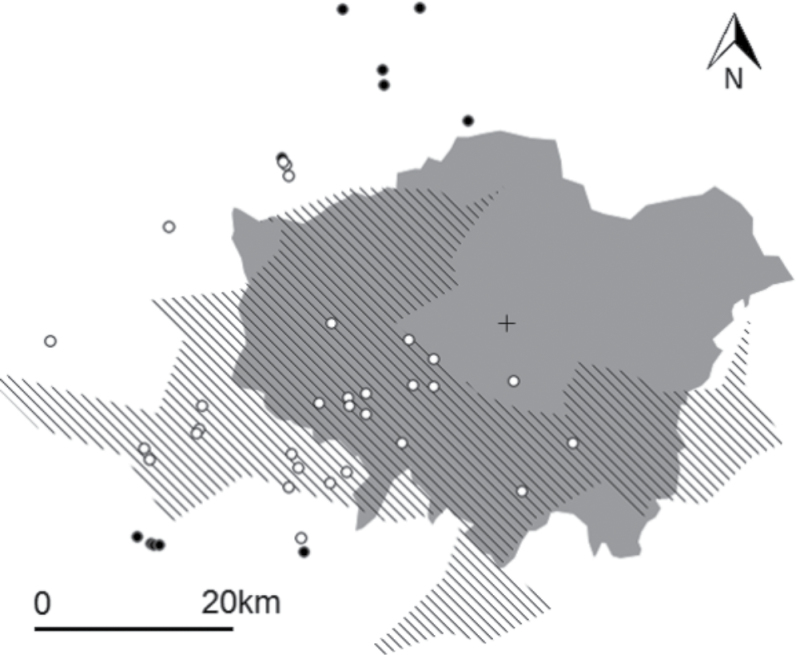
Experimental feeding site locations across London. Black circles represent parakeet free sites, white circles represent sites where parakeets were present, the solid gray area represents the area of Greater London, the lined polygon represents the area of the 2009 parakeet range (this is the extent of the Breeding Bird Survey 1 km^2^ squares where parakeets were recorded present in 2009). The cross represents the location of St. Paul’s Cathedral (sites were chosen within a 50-km radius of this).

Behavioral experiments were performed at 41 sites within a 50-km radius of the center of London, UK, using the London Natural History Society method of using St. Paul’s Cathedral as an approximate for the center of London (Figure 1). Sites comprised the gardens of members of the public who responded to advertisements made through the media, project website (www.projectparakeet.co.uk no longer online), local bird watching groups, and word of mouth and therefore the location of sites used were constrained by those offered, but still provided an adequate number of sites (*n* = 41) spread across a broad area of London and the surrounds (Figure 1). Procedures for contacting members of the public were assessed and passed by Imperial College’s research ethics committee in April 2010. Sites were at least 200 m apart (closest distance between 2 sites 226 m, see Figure 1) to minimize the risk of repeating the experiment on same individual native birds and were gardens that used bird feeders regularly to minimize the time needed for habituation of the birds to the feeding station. To test for potential habituation, gardens were classified as being either within or outside the current range of the invasive parakeet population. This was classified based on residents’ information and verification by observations made during the experiment. Parakeet absence was also verified by British Breeding Bird Survey parakeet presence–absence data for London and the Home Counties for 2009 (see Figure 1). The range of the great spotted woodpecker covers the entire area of the study (Balmer et al. 2013) and garden owners verified that the species visited each garden used in the experiment regularly, therefore it was considered that garden birds’ exposure to great spotted woodpeckers would be similar across all sites.

### Experimental procedure

A standardized feeding station was set up at each of the sites, with the behavior of free-living native birds being observed under a range of experimental and control conditions. Feeding stations comprised 2 squirrel proof feeders (The Nuttery NT28 and NT22 ASIN: B0007LQ3WQ, dimensions 20.3×20.3×34.3cm) hung on a steel shepherd hook pole Gardman (ASIN: B001F36RA8, height 218cm). Feeders were placed approximately 200cm above the ground as parakeets generally feed in plants and trees above ground (Clergeau and Vergnes 2011) and therefore any species affected by their presence are more likely to be species which also feed at this level rather than ground feeding species. Sunflower and peanuts were selected for use as the food sources as they are known to attract both parakeets and a variety of other birds species and therefore provided a high value food source likely to attract a variety of bird species (Lack 1986; Cowie and Simons 1991; Tvardíková and Fuchs 2010; Bonter et al. 2013). Sites were located in positions that could be viewed from a hidden location in order to be able to view the experiment without disturbing visiting birds. The feeding station was also placed in an area that had enough space for the experimental equipment (i.e., room for both the feeding station and the camera and tripod) and that was not obscured by vegetation or other structures. Where possible the feeding station was placed where the garden’s previous feeding station had been so as to limit the need for habituation to the new feeding station. To allow habituation of the local birds to the feeders before the experiment was conducted, each feeder was supplied with peanuts and sunflower seeds respectively for 2 weeks. This period was similar to acclimatization periods used for other bird studies (e.g., Sol et al. 2011; Orros and Fellowes 2012). These feeders represented a localized, high value resource that could be standardized across sites independent of season and prevented interference from gray squirrels, which are the only other nonnative vertebrate species present in the site locations that use garden bird feeders.

The behavioral experiments were conducted from May 2010 to February 2011 by H.L.P. or H.E.P., using 7 treatments that were designed to vary in the extent to which native birds were exposed to the presence of parakeets and control treatments. These consisted of 4 control treatments, which were an empty cage, a cage with a great spotted woodpecker in it, a cage with a great spotted woodpecker in it with a recording of a great spotted woodpecker call playing and an empty cage with just the call playing. In addition, there were 3 experimental treatments, these were a cage with a rose-ringed parakeet, a cage with a rose-ringed parakeet with a parakeet call playing, and an empty cage with just the parakeet call playing (see Table 1).

**Table 1:** Treatments used per site, “cage” refers to whether an experimental cage was empty or contained a live woodpecker or parakeet and “call” refers to whether there was no audio recording or if a recording of a woodpecker or parakeet call was played

			Cage	Call
Control 1	(C1)		Empty	None
Control 2	(C2)	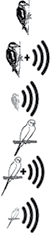	Woodpecker	None
Control 3	(C3)		Woodpecker	Woodpecker
Control 4	(C4)		Empty	Woodpecker
Treatment 1	(T1)		Parakeet	None
Treatment 2	(T2)		Parakeet	Parakeet
Treatment 3	(T3)		Empty	Parakeet

The order of the 7 treatments was randomized in each site.

Both a caged live parakeet and a recording of its call were used as treatments, as birds are known to be sensitive to both sight and sound (Sturkie 2000). In addition to the control of an empty cage with no recording it was necessary to also use native controls, making it possible to test for the strength of any effect of neophobia or habituation to the parakeet.

The great spotted woodpecker was chosen as a control species because it is the closest species in size that regularly feeds from hanging garden feeders, (rose-ringed parakeet: 400mm length, mass 120g; great spotted woodpecker: 220mm in length, mass 85g [Snow et al. 1998]) and its distribution overlaps with the study area (Balmer et al. 2013). Although smaller than the parakeet, this species has been observed to be aggressive at feeding stations in comparison to other birds and has been recorded successfully supplanting parakeets from feeding stations (Pithon 1998).

The 7 treatments were presented in a randomized order for 20min each (i.e., a total treatment time of 140min per site) generated through nonreplacement sampling in R (R Core Development Team 2009) for each individual site. For all treatments the cages (Montana KT3001, GTIN 04038374320048 46×63×53cm) were equipped with 2 metal bowls containing peanuts and water. For each treatment, the cage was placed on a stand at 0.3 m from the feeder station at the same height as the feeder for 20min. In the treatments involving sound, calls were played from a Ministry of Sound MOSMP020 MP3 player connected to 2 Skytronic 100.165 monitor speakers (frequency response 80–15000 Hz) played at full volume of an approximate amplitude of 80 dB(A), positioned on the ground directly below the cage. The great spotted woodpecker call was obtained from prerecorded material (Sample 1996), whereas the parakeet call was recorded from a series of vocalizations of an adult male rose-ringed parakeet in a garden in Richmond, Surrey, specifically for the study using a Sony Dictaphone. Both calls were general contact calls. Both calls were edited using Audacity 1.2.6 to minimize any background noise and repeated with intervals of random length (0–5 s) between calls to be of similar length (under 3min). These were played on repeat for the duration of the treatment.

The rose-ringed parakeet and great spotted woodpecker pairs (a male and female of each) used in the experimental trials were caught from the wild using a standard mist net under license from the British Trust for Ornithology and kept under Natural England (NE) licence (number 20101145) in an outside aviary between experiments. Aviaries were provisioned with nest boxes, ad libitum food and water, and provided sufficient room for flight. Each bird in a pair was used in alternate experiments in order to minimize stress and to control for any differences in behavior of visiting birds in response to differences in appearance due to sexual dimorphism. After all experiments were completed, the woodpeckers were given a 2-week soft-release at the catching site with open access to the aviary for food, water, and shelter. The parakeets were re-homed in captivity as required by the NE licence.

### Data collection

The activity of native birds at the feeder was recorded using a small camcorder (Panasonic SDR-S156) mounted on a tripod 3 m from the feeders. Video recordings of each trial were watched subsequently to record each visiting bird’s species, duration of visit (in seconds), whether or not feeding occurred, and which food was eaten (sunflower seed or peanut). For sites within the current range of the parakeet population, visits made by wild parakeets to the feeding station during the trials were removed from the data before analysis (this included 136 visits altogether and occurred in 12 out of the 30 sites within the parakeet range). Data were recorded by trained volunteer research assistants, using standardized methods. Error checking was carried out by double checking a random 5-min sample of each video.

Environmental conditions of the feeder location for each trial were recorded to take into account any variables that might affect foraging behavior and visiting activity of birds within each location. These included; feeder position in the sun or shade as direct sunlight can affect perception of risk and therefore foraging (Fernández-Juricic and Tran 2007), cloud cover and rain (cloudy, rain, or clear sky) as rainfall can also affect foraging (Hilton et al. 1999), wind strength (0–3: no wind to strong wind) which has been found to affect visiting rates (Cowie and Simons 1991), time of year measured as months from May (1–10) to account for seasonal effects on feeding requirements (Chamberlain et al. 2005), time of day (AM or PM) to account for changes in foraging activity during the day (Bonter et al. 2013), and distance of the feeding station to vegetation cover (<1 m, 1–2 m, >3 m) as distance to cover is known to affect foraging in birds (Cowie and Simons 1991). Distance of the site from the center of London (measured in km from St. Paul’s Cathedral) was also measured and used to account for any between site differences due to differences in the level of urbanization in the surrounding area (Crawley 2011), which might be confounded with parakeet distribution.

### Data analysis

The data on the behavior of native birds at the feeding stations were analyzed to test for differences between experimental treatments in a number of dependent variables: model 1 tested the number of visits by native birds to the feeders; model 2 tested the proportion of visits that included a bout of feeding; model 3 tested the absolute time spent feeding within a visit; model 4 tested the proportion of time spent vigilant during a feeding visit. These were chosen to test for whether parakeets inhibited birds from visiting (model 1) and then whether and how they affected foraging success (models 2 and 3) and the trade-off between foraging and vigilance (model 4). Vigilance is defined here as the proportion of time spent not feeding during feeding visits to peanuts and is an indication of risk perception. The absolute time feeding is likely to be correlated with the amount of food taken and therefore an indication of foraging success. Behaviors other than feeding and vigilance, such as aggression or preening were extremely rare and so the assumption was made that when birds are on the feeding station and not feeding then they were being vigilant. Birds visiting the feeders were not individually marked and so multiple visits by the same individual could not be accounted for but all sites were treated equally. In addition to the experimental treatments described in Table 1, we also tested for an effect of whether or not the site was within the range of the invasive parakeet population.

Data were analyzed using generalized linear models in R (R Core Development Team 2009), using the lme4 package (Bates et al. 2012). Models 1 and 3 were over dispersed so these included an observation-level random effect and were fitted using a Poisson log-normal error structure and a log link function (Elston et al. 2001). Models 2 and 4 were fitted using a binomial error structure and logit link function. Model 1 was analyzed using a data subset of the summed total visits for each treatment per site. Model 2 was analyzed using the full data set of each individual visit. Sunflower seeds were visited for such a short time per visit (median = 1 s, interquartile range [IQR] = 1, *n* = 2117) regardless of treatment, that it was not possible to analyze differences in time spent feeding. Therefore, only feeding visits to peanuts were analyzed for differences in time feeding, so models 3 and 4 were analyzed using a subset of only feeding visits where the bird fed on peanuts.

In models the experimental feeding site identification was added as a random effect. Variables measured in each site were added into each model as fixed effects: inside or outside the parakeet range, cloud cover and rain, wind strength, sun or shade, time of day (AM or PM), time of year (number of months from May), distance of the feeding station to vegetation, and distance (km) from central London. Inside or outside the parakeet range was categorized as binomial (absent or present), as preliminary analysis using a continuous measure of parakeet numbers in the site ranging from 0 to 5 found no difference in visits between the sites ranging from 1 to 5. The order in which each treatment took place were also added as a fixed effect to account for any interference that may result in a time lag between exposure to a treatment and resumption of foraging (Sih 1992; Evans 2003). For models 2, 3, and 4 the species visiting the feeding station were also added as a fixed effect to account for differences in behavior between species in response to the treatments. This variable could not be tested on model 1 due to it being a measure of total visits and therefore excluding details of individual visits. No explanatory variables included in the full models were highly correlated (*r* < 0.249 in all cases) see Supplementary Table S6.

Following Crawley (2013), we estimated minimum adequate models by entering all fixed effects and dropping them sequentially until only those that explained significant variation remained (see site variable effect results in Supplementary Table S4). At each stage the reduced model was tested against the previous model to check that a significant amount of variation had not been lost using a chi-squared statistic in an Anova.

## RESULTS

In total, at the 30 sites inside the current range of the parakeet population, the feeding station was visited 6872 times (median = 96, IQR = 42–252 visits per site) and in the 11 sites outside the parakeet range the feeding station was visited 4021 times (149, 72–311). Across these visits 18 native bird species were observed on the feeding stations, which were predominantly blue tits (*Cyanistes caeruleus*, 42% of visits) and great tits (*Parus major*, 41%) (a summary of visits made by each species, Supplementary Table S5).

### Sites inside the current range of the parakeet population

**Figure 2:**
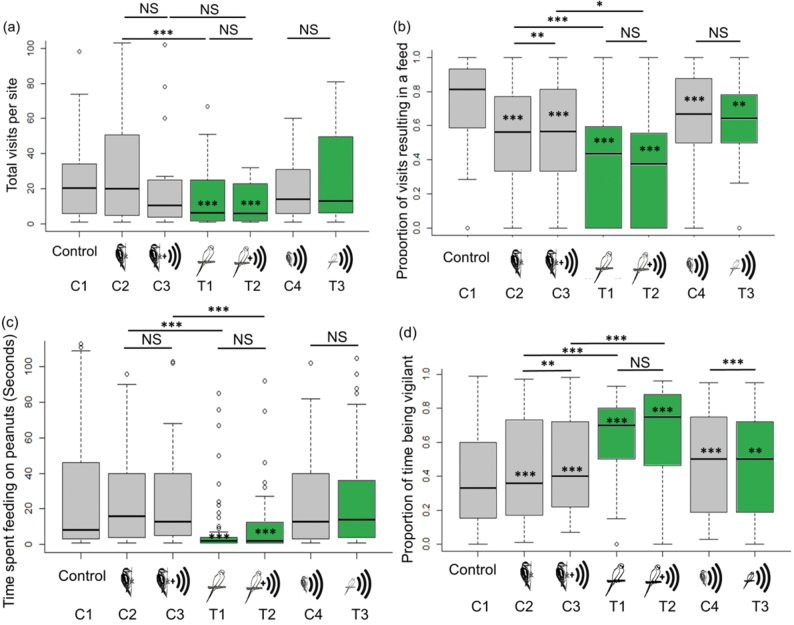
Box and whisker plots for (a) number of visits (*n* = 6826), (b) proportion of visits resulting in a feeding event (*n* = 6826), (c) time spent feeding (seconds) on peanuts per feeding visit (*n* = 555), (d) vigilance (proportion of time spent not feeding [seconds] per feeding visit to peanuts) (*n* = 555), per treatment for sites inside the parakeet range (*n* = 30). Significant values within a box refer to the difference of the treatment from the control (C1), values outside a box and on a solid line refer to between treatments, (*P* values, ***P < 0.001, **P < 0.01, *P < 0.05). Gray boxes show controls and green boxes show treatments. C4 and T3 are both call only conditions and so are grouped together on the right of each panel to aid comparison.

Our analyses demonstrated that at sites inside the current range of the parakeet population, experimental exposure to caged parakeets resulted in a significant reduction in the number of visits by native birds to the feeders (Figure 2a), a significant reduction in the number of visits that included a feeding bout (Figure 2b), a significant reduction in the absolute time spent feeding (Figure 2c), and a significant increase in the proportion of time spent vigilant (Figure 2d), (Supplementary Table S1). For all these behaviors, changes in the same direction were also observed in the presence of a woodpecker (Figure 2, Supplementary Table S1), but the changes in the presence of a parakeet were significantly greater than those in the presence of a woodpecker (Figure 2, Supplementary Table S2).

A parakeet call alone reduced the proportion of visits resulting in a feeding bout and increased the proportion of time spent vigilant (Figure 2, Supplementary Table S1). This effect was significantly greater from that of the woodpecker call for the proportion of time spent vigilant. But in all cases the effect of the parakeet call alone was less pronounced than that of the presence of a caged parakeet (Figure 2, Supplementary Table S2) and the addition of a parakeet call to the presence of a parakeet had little additional effect for all response behaviors (Figure 2, Supplementary Table S2).

### Sites outside the current range of the parakeet population

**Figure 3:**
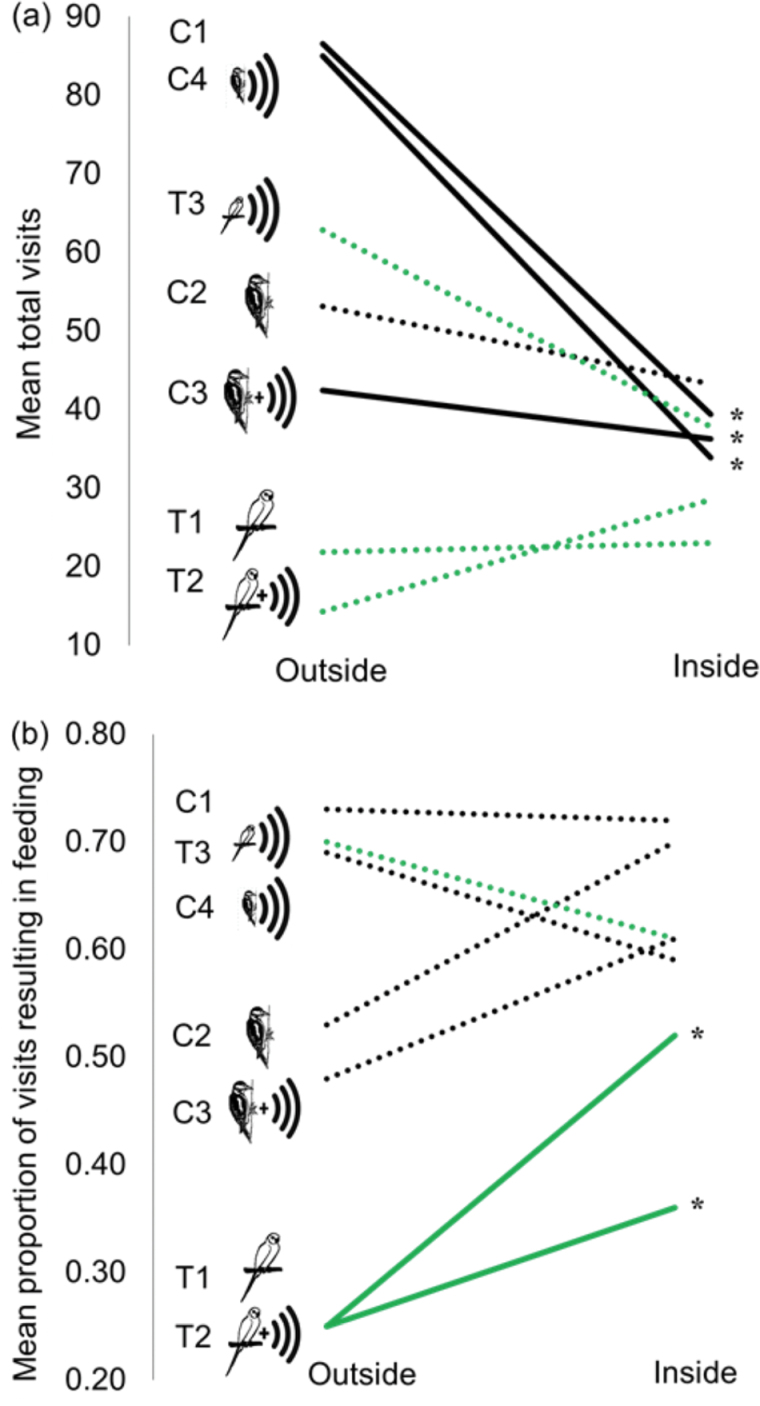
Differences between sites per treatments outside and inside parakeet range for (a) mean total visits, and (b) the proportion of visits resulting in a feed (visits outside range *n* = 4027, inside range *n* = 6826). Black lines show controls and gray lines show treatments. Dotted lines denote differences between range sites within treatments which are not significant, solid lines are significant (*P* values, *P < 0.05). Significant difference refers to differences within treatments between sites outside and inside the parakeet range.

Overall the patterns in response to treatments were consistent with those inside the current parakeet range (Supplementary Tables S1 and S2). The number of total visits to feeding stations was higher for 3 out of 4 of the control treatments (C1, C3, and C4) at sites outside the parakeet range compared with sites within (Figure 3a). However, notably, parakeet treatments (T1 and T2) resulted in a lower proportion of feeding events outside of the parakeet range than inside (Figure 3b and Supplementary Table S3). During the 2 caged parakeet treatments there were so few feeding visits to peanuts, (T1, *n* = 7; T2 *n* = 3) that changes found for total feeding time and vigilance, in response to these 2 treatments could not be confidently compared (Supplementary Tables S1–S3). However, the parakeet call alone treatment (T3), which did have enough visits to compare (visits *n* = 95 outside, *n* = 63 inside), was shown to elicit a stronger vigilance response inside the parakeet range compared with outside (T3 outside range vs T3 inside range; Supplementary Table S3).

## DISCUSSION

Our findings show that experimental exposure to parakeets influences the behavior of native birds, resulting in reduced feeding and increased vigilance. These changes in behavior are much more pronounced in the presence of a parakeet than in the presence of a dominant native species, the great spotted woodpecker. While visit rates drop significantly in the presence of a parakeet both inside and outside the current parakeet range, visits that do occur are more likely to result in feeding inside the range. Taken together, these results suggest that interference competition between a nonnative species and the native fauna does appear likely in this study system, and that some habituation may occur in the native populations.

### Interference competition

Interspecific interference competition between native and nonnative fauna is a concern as it may lead to reduced energy intake, and thus potentially lower the fitness of native birds (Gustafsson 1987; Cresswell 2008). Similarly, increased vigilance induced by the presence of nonnative species can diminish the relative value of a food resource through increasing access costs (Cooper and Frederick 2007).

The majority of visits to the feeding stations were by blue tits and great tits. These species are common, ubiquitous birds in urban areas in the United Kingdom (Cowie et al. 1988; Balmer et al. 2013). Interspecific competition between tit species has been shown to cause displacement of individuals of less dominant species to lower quality food sources in coniferous forest (Alatalo et al. 1987), and spatial displacement and niche compression of blue tits in oak woodland (Herrera 1978). The presence of parakeets may simply result in temporary displacement of native species from a food source, with minimal costs. Temporal niche shift behavior has been shown in the timing of great tit dawn singing in response to supplemental feeding (Saggese et al. 2011) and also in invasive mink (*Neovison vison*) avoiding 2 native mustelid species (*Lutra lutra* and *Mustela putorius*) during foraging (Harrington et al. 2009). Our analyses did, however, control for time of day and found it was not a significant predictor of the number of visits to the feeding station. This suggests that parakeets may induce a spatial, rather than, temporal shift in native bird foraging behavior. Similar spatial shifts in response to environmental changes, such as loss of access to food resources, have been shown to lead to reduced population sizes (Durell et al. 2005, 2006; Stillman and Goss-Custard 2010). Consistent displacement of native birds from high-quality resources may, therefore, be expected to have long-term implications for native species’ populations.

There are very few examples in the literature of dominance over food sources by nonnative species resulting in displacement of native species. Examples of this occurring with nonnative vertebrates include an invasive gecko (*Lepidodactylus lugubris*) in Hawaii (Petren and Case 1996) and the invasive gray squirrel (*Sciurus carolinensis*) in the United Kingdom (Kenward and Holm 1993) but see (Wauters and Gurnell 1999). The subtle changes in feeding behavior seen in response to parakeet presence may represent a mechanism for displacement, which to our knowledge would be the first case of such by a nonnative avian species and therefore merits further investigation.

### Habituation

Our finding of a higher likelihood of feeding in the presence of a parakeet within the parakeet range compared with sites outside, suggests habituation to parakeets following prior exposure. This is particularly evident considering the overall lower mean total number of visits in the sites within the parakeet range compared with those outside. Without data on individual behavior, it is not possible to distinguish whether this effect is due to an increased number of visits by bold individuals who have become accustomed to the parakeets, comparable to behavior seen in several other bird species exposed to a predator (Quinn and Cresswell 2005; van Oers 2005; Minderman et al. 2010; Rockwell et al. 2012), but see Couchoux and Cresswell (2011); or, if the perception of risk lowers for all individuals with continued exposure (Ellenberg et al. 2009; Rodríguez-Prieto et al. 2011). The former would suggest that some individuals may be disproportionately impacted by nonnative species’ presence, whereas the latter would indicate population-wide adjustment to the presence of a nonnative species, and potentially lower overall impact.

In contrast to habituation to the presence of the parakeets, we also found some evidence of reinforcement behavior, such that native birds within the parakeet range were more vigilant when exposed to a parakeet call, which suggests that prior exposure to parakeet calls has an influence on behavior. This finding is consistent with previous studies demonstrating this initial lack of response of prey to the calls of a novel avian predator (Reudink et al. 2007; Elmasri et al. 2012) and studies demonstrating learned association of predator cues (reviewed by Griffin et al. 2000).

It is possible differences in native bird behavior between sites within and outside the parakeet range are due to differences associated with urbanization (Lowry et al. 2013). For example, bird species in urban areas, including blue tits and great tits, have been demonstrated to have differing behavioral adaptation to predators compared with rural areas (Møller and Ibáñez-Álamo 2012) and urban populations of song sparrow (*Melospiza melodia*) have been found to be bolder and show greater territorial aggression compared with rural populations (Evans et al. 2010). We did control for an urbanization effect in our analysis, by testing for an effect of distance from the city center, which was not found to be of importance. Regardless of the causes for the differences in response of birds inside and outside the parakeet range, the higher proportion of feeding visits at sites inside the range in the presence of a parakeet was still lower than the control treatments and therefore foraging visits were still less successful across all sites in the presence of a parakeet at a food source, despite the prior exposure. This indicates that the inhibition of foraging in the presence of a parakeet is a permanent effect and not just a case of neophobia.

### Ecological implications

It should be noted that wild rose-ringed parakeets often forage gregariously and therefore monopolize a feeding site (Pithon 1998). During our experiment, 95 out of 136 visits by wild parakeets to the experimental feeding station were when one of our captive parakeets was present in the cage, further demonstrating parakeets’ gregarious nature. Given this we would expect that the impacts demonstrated here are a conservative estimate of those that would be seen with free-living parakeets.

It has been hypothesized that optimal foraging in urban settings can alter the community structure and result in biodiversity loss (Shochat et al. 2004). It is also common for invading species to establish where competition among the resident species is low (Crawley et al. 1986) and so the dominance of nonnative species can also be increased by the provision of artificial feeding stations in gardens (Chace and Walsh 2006; McKinney 2006; Jones and Reynolds 2008; Cannon 2010). The link between garden feeding and the success of invasive species is seen directly in Chicago, where the persistence and growth of the monk parakeet (*Myiopsitta monachus*) population is attributed to the generalist diet of the nonnative species and its sole use of bird feeders in winter (South and Pruett-Jones 2000). Clearly garden food provisioning plays a major role in the persistence of invasive populations, which in tandem with the temporally stable climate and homogenization of the environment makes the urban landscape favorable to nonnative species (Garden et al. 2006). Given our evidence for foraging disruption at artificial food sources and the potential reliance of the parakeets on garden bird feeders (Clergeau and Vergnes 2011), the exclusion of parakeets from access to garden bird feeders might benefit native species through reducing the interference competition. In addition, exclusion of parakeets would simultaneously reduce the foraging benefits of urban areas for the parakeets, which may in turn limit the persistence of the parakeet populations. Therefore, an interesting implication of this research is that exclusion of parakeets from garden bird feeders may provide an option for mitigating impacts of parakeets on garden birds.

In conclusion, we provide evidence demonstrating interference foraging competition between a nonnative bird and 2 native birds common to urban areas. While this study does not provide proof of any population level change as a result of the disrupted foraging, it does demonstrate a mechanism by which nonnative birds could potentially impact native species at the population level and shows a need for further investigation. The increased establishment of rose-ringed parakeets in the United Kingdom (Tayleur 2010) and across Europe (Strubbe and Matthysen 2008) as well as other nonnative bird species in urban areas across the world (Peacock et al. 2007; Blackburn et al. 2009; Bonter et al. 2010), highlights the potential for widespread occurrence of similar effects of foraging behavior on urban birds.

## SUPPLEMENTARY MATERIAL

Supplementary material can be found at http://www.beheco.oxfordjournals.org/

## FUNDING

This work was supported by a research studentship from the Biotechnology and Biological Sciences Research Council (reference BB/D526410/1) to H.L.P. and by The Nuttery Ltd.

## Supplementary Material

Supplementary Data
