# Supplementary material for: Experimental evidence of impacts of an invasive parakeet on foraging behavior of native birds
Source: Behav Ecol. 2014 Mar 7;25(3):582–90. doi: 10.1093/beheco/aru025 (PMC4014307; doi:10.1093/beheco/aru025)
Supplement: Supplementary Data [file supp_25_3_582__index.html]

Experimental evidence of impacts of an invasive parakeet on foraging behavior of native birds — Experimental evidence of impacts of an invasive parakeet on foraging behavior of native birds — Supplementary Data 

# Experimental evidence of impacts of an invasive parakeet on foraging behavior of native birds

## Supplementary Data

Data files

**Files in this Data Supplement:**

- Supplementary Data - Supplementary Data
