# Supplementary material for: Experimental evidence of impacts of an invasive parakeet on foraging behavior of native birds
Source: Behav Ecol. 2014 Mar 7;25(3):582–90. doi: 10.1093/beheco/aru025 (PMC4014307; doi:10.1093/beheco/aru025)
Supplement: Supplementary Data [file supp_aru025_Supplementary_Material.docx]

Supplementary material

Table S1 Mean values for measured response behaviours per treatment for sites outside the parakeet range (n=11) and sites inside the parakeet range (n=30) and corresponding differences between each treatment and control one (C1) (GLMM z-values, with significant p-values, ***P<0.001, **P<0.01, *p<0.05). # denotes results which are unreliable due to too few data points to make a comparison.

|  |  | Sites outside parakeet range | | | | | Sites inside parakeet range | | | |
| --- | --- | --- | --- | --- | --- | --- | --- | --- | --- | --- |
| Model | Behaviour | Treatment | mean | *±*SE | n | z and p values | mean | *±*SE | n | z and p values |
| 1 | Total visits | C1 | 86.55 | 17.99 | 952 |  | 39.4 | 11.59 | 1117 |  |
|  |  | C2 | 53.18 | 10.82 | 585 | -1.68 | 43.25 | 11.52 | 1226 | 0.043 |
|  |  | C3 | 42.45 | 7.92 | 467 | -1.960 * | 36.24 | 11.08 | 1030 | -1.846 |
|  |  | T1 | 21.82 | 7.45 | 240 | -5.288 *** | 23 | 7.98 | 616 | -3.691 *** |
|  |  | T2 | 14.27 | 5.35 | 157 | -6.546 *** | 28.5 | 14.74 | 733 | -3.634 *** |
|  |  | C4 | 85 | 21.63 | 935 | -0.632 | 33.9 | 9.69 | 1012 | -0.394 |
|  |  | T3 | 62.82 | 15.5 | 691 | -1.602 | 37.82 | 10.20 | 1092 | -0.31 |
| 2 | Proportion  feeding visits | C1 | 0.73 | 0.014 | 952 |  | 0.72 | 0.013 | 1117 |  |
|  |  | C2 | 0.53 | 0.021 | 585 | -7.858 *** | 0.7 | 0.013 | 1226 | -5.788 *** |
|  |  | C3 | 0.48 | 0.023 | 467 | -8.613 *** | 0.61 | 0.015 | 1030 | -8.586 *** |
|  |  | T1 | 0.25 | 0.028 | 240 | -12.222 *** | 0.52 | 0.02 | 616 | -12.119 *** |
|  |  | T2 | 0.25 | 0.035 | 157 | -9.755 *** | 0.36 | 0.018 | 733 | -10.676 *** |
|  |  | C4 | 0.7 | 0.015 | 935 | -3.871 *** | 0.61 | 0.015 | 1012 | -3.065 *** |
|  |  | T3 | 0.69 | 0.018 | 691 | -3.332 *** | 0.59 | 0.015 | 1092 | -5.076 ** |
| 3 | Time feeding | C1 | 63.12 | 6.66 | 104 |  | 36.43 | 6.1 | 83 |  |
|  |  | C2 | 41.22 | 7.55 | 37 | -2.183 * | 30.65 | 4.29 | 86 | -0.612 |
|  |  | C3 | 87.24 | 19.49 | 38 | -1.748 | 27.18 | 4.93 | 45 | -0.438 |
|  |  | T1 | 30.43 | 17.32 | 7 | #-2.330 * | 5.75 | 1.36 | 105 | -6.161 *** |
|  |  | T2 | 11.33 | 10.33 | 3 | #-2.802 ** | 14.24 | 4.61 | 55 | -4.732 *** |
|  |  | C4 | 51.24 | 7.17 | 75 | -1.285 | 35.31 | 6.2 | 86 | -1.309 |
|  |  | T3 | 52.32 | 7.06 | 63 | -2.114 * | 29.12 | 4.4 | 95 | -1.309 |
| 4 | Proportion time  vigilant | C1 | 0.31 | 0.028 | 104 |  | 0.397 | 0.031 | 83 |  |
|  |  | C2 | 0.481 | 0.052 | 37 | 16.96 *** | 0.428 | 0.032 | 86 | 11.582 *** |
|  |  | C3 | 0.394 | 0.051 | 38 | 9.85 *** | 0.453 | 0.042 | 45 | 13.366 *** |
|  |  | T1 | 0.694 | 0.124 | 7 | #32.06*** | 0.63 | 0.024 | 105 | 21.069 *** |
|  |  | T2 | 0.7 | 0.115 | 3 | # 5.78 *** | 0.643 | 0.038 | 55 | 18.622 *** |
|  |  | C4 | 0.448 | 0.036 | 75 | 20.15 *** | 0.494 | 0.032 | 86 | 12.358 *** |
|  |  | T3 | 0.407 | 0.041 | 63 | 5.44 *** | 0.474 | 0.030 | 95 | 18.146 *** |

Table S2 Differences in response behaviours between treatments other than C1 for both sites outside (n=11) and inside (n=30) the parakeet range (GLMM z-values, with significant p-values, ***P<0.001, **P<0.01, *p<0.05). # denotes results which are unreliable due to too few data points to make a comparison.

| Model | Behaviour | Treatment | Sites outside range  z and p values | Sites inside range  z and p values | Model | Behaviour | Treatment | Sites outside range  z and p values | Sites inside range  z and p values |
| --- | --- | --- | --- | --- | --- | --- | --- | --- | --- |
| 1 | Total visits | C2 v T1 | -3.650*** | -3.725*** | 3 | Time feeding | C2 v T1 | -1.211 | -5.888*** |
|  |  | C3 v T2 | -4.648*** | -1.796 |  |  | C3 v T2 | -2.204* | -3.852*** |
|  |  | C2 v C3 | -0.273 | -1.874 |  |  | C2 v C3 | 0.335 | 0.087 |
|  |  | T1 v T2 | -1.280 | -0.017 |  |  | T1 v T2 | #-1.106 | 0.634 |
|  |  | C4 v T3 | -0.967 | 0.077 |  |  | C4 v T3 | -0.925 | -0.170 |
|  |  | T1 v T3 | 3.712*** | 3.336*** |  |  | T1 v T3 | #1.445 | 5.353*** |
|  |  | C2 v C4 | 1.047 | -0.435 |  |  | C2 v C4 | 1.097 | -0.713 |
| 2 | Proportion of  feeding visits | C2 v T1 | -6.608*** | -7.391*** | 4 | Proportion of time vigilant | C2 v T1 | 18.864*** | 12.163*** |
|  |  | C3 v T2 | -3.970*** | -2.323* |  |  | C3 v T2 | 3.665*** | 5.936*** |
|  |  | C2 v C3 | -1.384 | -3.081** |  |  | C2 v C3 | -7.490*** | 2.912** |
|  |  | T1 v T2 | 0.512 | 1.711 |  |  | T1 v T2 | #-5.910*** | -1.487 |
|  |  | C4 v T3 | 0.341 | -1.925 |  |  | C4 v T3 | -12.169*** | 5.595*** |
|  |  | T1 v T3 | 9.784*** | 7.640*** |  |  | T1 v T3 | #-28.160*** | -8.069*** |
|  |  | C2 v C4 | -4.280*** | -2.666** |  |  | C2 v C4 | -1.938 | 0.094 |

Table S3 Differences in response behaviours between sites outside (n=11) and inside (n=30) the parakeet range (GLMM z-values, with significant p-values, **P<0.01, *P<0.05). # denotes results which have too few data points to be confident in the comparison.

| \| Model \| Behaviour \| Treatment \| Difference \| SE \| z-value \| P-value \|  \| Model \| Behaviour \| Treatment \| Difference \| SE \| z-value \| P-value \| \| \| --- \| --- \| --- \| --- \| --- \| --- \| --- \| --- \| --- \| --- \| --- \| --- \| --- \| --- \| --- \| --- \| \| 1 \| Total visits \| C1 \| -1.183 \| 0.460 \| -2.571 \| 0.010 \| * \| 3 \| Time feeding \| C1 \| -0.387 \| 0.298 \| -1.299 \| 0.194 \|  \| \| \|  \|  \| C2 \| -0.642 \| 0.468 \| -1.371 \| 0.170 \|  \|  \|  \| C2 \| 0.067 \| 0.341 \| 0.198 \| 0.843 \|  \| \| \|  \|  \| C3 \| -0.963 \| 0.472 \| -2.041 \| 0.041 \| * \|  \|  \| C3 \| -0.024 \| 0.383 \| -0.062 \| 0.951 \|  \| \| \|  \|  \| T1 \| -0.260 \| 0.482 \| -0.54 \| 0.589 \|  \|  \|  \| #T1 \| -0.555 \| 0.621 \| -0.894 \| 0.371 \|  \| \| \|  \|  \| T2 \| 0.182 \| 0.487 \| 0.373 \| 0.709 \|  \|  \|  \| #T2 \| 0.762 \| 0.925 \| 0.824 \| 0.410 \|  \| \| \|  \|  \| C4 \| -1.066 \| 0.463 \| -2.304 \| 0.021 \| * \|  \|  \| C4 \| -0.414 \| 0.307 \| -1.349 \| 0.177 \|  \| \| \|  \|  \| T3 \| -0.742 \| 0.466 \| -1.593 \| 0.111 \|  \|  \|  \| T3 \| -0.198 \| 0.309 \| -0.641 \| 0.522 \|  \| \| \| 2 \| Proportion of feeding visits \| C1 \| -0.167 \| 0.228 \| -0.735 \| 0.462 \|  \| 4 \| Proportion of time vigilant \| C1 \| 0.292 \| 0.399 \| 0.730 \| 0.465 \|  \| \| \|  \| C2 \| 0.216 \| 0.227 \| 0.953 \| 0.341 \|  \|  \| C2 \| 0.063 \| 0.399 \| 0.158 \| 0.874 \|  \| \| \|  \|  \| C3 \| 0.069 \| 0.236 \| 0.291 \| 0.771 \|  \|  \|  \| C3 \| 0.766 \| 0.401 \| 1.910 \| 0.056 \|  \| \| \|  \|  \| T1 \| 0.607 \| 0.271 \| 2.241 \| 0.025 \| * \|  \|  \| #T1 \| -0.971 \| 0.408 \| -2.380 \| 0.017 \| * \| \| \|  \|  \| T2 \| 0.700 \| 0.296 \| 2.365 \| 0.018 \| * \|  \|  \| #T2 \| 0.398 \| 0.466 \| 0.854 \| 0.393 \|  \| \| \|  \|  \| C4 \| -0.042 \| 0.227 \| -0.187 \| 0.852 \|  \|  \|  \| C4 \| 0.186 \| 0.398 \| 0.467 \| 0.641 \|  \| \| \|  \|  \| T3 \| -0.307 \| 0.229 \| -1.340 \| 0.180 \|  \|  \|  \| T3 \| 1.106 \| 0.398 \| 2.777 \| 0.005 \| ** \| \| |  |
| --- | --- | --- | --- | --- | --- | --- | --- | --- | --- | --- | --- | --- | --- | --- | --- | --- | --- | --- | --- | --- | --- | --- | --- | --- | --- | --- | --- | --- | --- | --- | --- | --- | --- | --- | --- | --- | --- | --- | --- | --- | --- | --- | --- | --- | --- | --- | --- | --- | --- | --- | --- | --- | --- | --- | --- | --- | --- | --- | --- | --- | --- | --- | --- | --- | --- | --- | --- | --- | --- | --- | --- | --- | --- | --- | --- | --- | --- | --- | --- | --- | --- | --- | --- | --- | --- | --- | --- | --- | --- | --- | --- | --- | --- | --- | --- | --- | --- | --- | --- | --- | --- | --- | --- | --- | --- | --- | --- | --- | --- | --- | --- | --- | --- | --- | --- | --- | --- | --- | --- | --- | --- | --- | --- | --- | --- | --- | --- | --- | --- | --- | --- | --- | --- | --- | --- | --- | --- | --- | --- | --- | --- | --- | --- | --- | --- | --- | --- | --- | --- | --- | --- | --- | --- | --- | --- | --- | --- | --- | --- | --- | --- | --- | --- | --- | --- | --- | --- | --- | --- | --- | --- | --- | --- | --- | --- | --- | --- | --- | --- | --- | --- | --- | --- | --- | --- | --- | --- | --- | --- | --- | --- | --- | --- | --- | --- | --- | --- | --- | --- | --- | --- | --- | --- | --- | --- | --- | --- | --- | --- | --- | --- | --- | --- | --- | --- | --- | --- | --- | --- | --- | --- | --- | --- | --- | --- | --- | --- | --- | --- | --- | --- | --- | --- | --- | --- | --- | --- | --- | --- | --- | --- | --- | --- | --- | --- | --- | --- | --- | --- | --- | --- | --- | --- |

Table S4 Variables (other than treatment and site distribution outside or inside the parakeet range) remaining in the minimal adequate models as fixed effects. The effect of each variable was tested by dropping it from the final model and comparing the final and reduced model by comparing their log-likelihoods. Variables which did not remain in any of the minimal adequate models include: distance to cover (m), wind strength, station in shade or sunlight, distance to centre of London (km).

| Model | Measured behaviour | Fixed effects | d.f | chi-squared | p-value |
| --- | --- | --- | --- | --- | --- |
|  |  |  |  |  |  |
| 1 | total visits | month (May to February, 1-10) | 17 | 4.21 | 0.040 |
|  |  | time of day (am/pm) | 17 | 30.82 | <0.001 |
|  |  |  |  |  |  |
| 2 | proportion of visits | order (1-7) | 35 | 47.37 | <0.001 |
|  | resulting in feed | time of day (am/pm) | 35 | 315.35 | <0.001 |
|  |  | Species | 17 | 1985.30 | <0.001 |
|  |  |  |  |  |  |
| 3 | time feeding | time of day (am/pm) | 25 | 17.17 | <0.001 |
|  |  | month (May to February, 1-10) | 25 | 14.36 | <0.001 |
|  |  | Species | 18 | 63.01 | <0.001 |
|  |  |  |  |  |  |
| 4 | vigilance | order (1-7) | 26 | 96.87 | <0.001 |
|  |  | time of day (am/pm) | 26 | 56.14 | <0.001 |
|  |  | weather (cloudy, sun, rain) | 25 | 25.71 | <0.001 |
|  |  | Species | 19 | 644.99 | <0.001 |

Table S5 Total number of visits and percentage of total visits of each species that visited the feeding stations within and outside the parakeet range. Visits by 16 of the species were too rare to confidently compare foraging response, but it was found that greats tits spent less time feeding (median = 4 seconds; IQR = 1- 19;  z = -7.58, p < 0.001) and more time being vigilant (median proportion of time = 0.566 seconds; IQR = 0.255-0.822; z = 16.47; p < 0.001) on peanuts than blue tits (time feeding: median = 18seconds; IQR = 3 – 58.5, proportion of time vigilant: median = 0.393; IQR = 0.157 -0.750).

| Species | Visits Within parakeet range | % total | Visits Outside parakeet range | % total |  |
| --- | --- | --- | --- | --- | --- |
| Great tit | 2876 | 41.85 | 1665 | 41.41 |  |
| Blue tit | 2950 | 42.93 | 1634 | 40.64 |  |
| Blackbird | 4 | 0.06 | 0 | 0 |  |
| Chaffinch | 3 | 0.04 | 0 | 0 |  |
| Coal tit | 170 | 2.47 | 92 | 2.29 |  |
| Carrion Crow | 1 | 0.01 | 0 | 0 |  |
| Goldfinch | 43 | 0.63 | 0 | 0 |  |
| Greenfinch | 33 | 0.48 | 7 | 0.17 |  |
| Great spotted woodpecker | 43 | 0.63 | 52 | 1.29 |  |
| House sparrow | 1 | 0.015 | 41 | 1.02 |  |
| Jay | 0 | 0 | 5 | 0.12 |  |
| Long tailed tit | 0 | 0 | 7 | 0.17 |  |
| Magpie | 2 | 0.03 | 6 | 0.15 |  |
| Nuthatch | 21 | 0.31 | 6 | 0.15 |  |
| Pigeon | 10 | 0.15 | 0 | 0 |  |
| Robin | 17 | 0.25 | 11 | 0.27 |  |
| Woodpigeon | 16 | 0.23 | 3 | 0.07 |  |
| Unidentified | 682 | 9.92 | 492 | 12.24 |  |
| Total | 6872 |  | 4021 |  |  |

Table S6 Collinearity scores for all continuous fixed effect variables in the full models, < 0.5 are considered highly correlated.

| Model | Measured behaviour | Fixed effects | order | month | to London |
| --- | --- | --- | --- | --- | --- |
| 1 | total visits |  |  |  |  |
|  |  | order (1-7) |  |  |  |
|  |  | month (May to February, 1-10) | 0.034 |  |  |
|  |  | distance to London (km) | 0.003 | -0.072 |  |
|  |  | wind strength (0-3) | -0.034 | -0.223 | 0.064 |
|  |  |  |  |  |  |
| 2 | proportion of visits | order (1-7) |  |  |  |
|  | resulting in feed | month (May to February, 1-10) | -0.013 |  |  |
|  |  | distance to London (km) | -0.004 | -0.158 |  |
|  |  | wind strength (0-3) | -0.033 | -0.3 | 0.146 |
|  |  |  |  |  |  |
| 3 | time feeding | order (1-7) |  |  |  |
|  |  | month (May to February, 1-10) | 0.001 |  |  |
|  |  | distance to London (km) | 0.004 | -0.311 |  |
|  |  | wind strength (0-3) | 0.048 | -0.327 | 0.249 |
|  |  |  |  |  |  |
| 4 | vigilance | order (1-7) |  |  |  |
|  |  | month (May to February, 1-10) | -0.013 |  |  |
|  |  | distance to London (km) | 0.004 | -0.195 |  |
|  |  | wind strength (0-3) | -0.005 | -0.126 | 0.078 |
